# Barriers in physical access to maternal health services in rural Ethiopia

**DOI:** 10.1186/s12913-015-1161-0

**Published:** 2015-11-04

**Authors:** Yemisrach B. Okwaraji, Emily L. Webb, Karen M. Edmond

**Affiliations:** Department of Public Health and General Practice, Faculty of Medicine, Norwegian University of Science and Technology, Trondheim, Norway; Department of Infectious Disease Epidemiology, Faculty of Epidemiology and Population Health, London School of Hygiene & Tropical Medicine, London, UK; School of Paediatrics and Child Health, University of Western Australia, Perth, Western Australia Australia

## Abstract

**Background:**

Identifying women with poor access to health services may inform strategies for improving maternal and child health outcomes. The aim of this study was to explore risk factors associated with access to health facilities (in terms of physical distance) among women of reproductive age (15–49 years) in Dabat district, a rural area of north-western Ethiopia.

**Methods:**

A randomly selected cross sectional survey of 1,456 rural households was conducted. Data were collected during home visits. Data on household assets and socio-demographic data (including age, education level, occupation, religion and ethnicity) were collected on 1,420 women. A geographic information system (GIS) was used to map locations of all households, the district health centre and the smaller health posts. Travel time from households to health facilities was estimated, incorporating information on the topography and terrain of the area. The primary outcomes were: 1) travel time from household to nearest health post 2) travel time from household to health centre. Analysis was conducted using multiple linear regression models and likelihood ratio tests.

**Results:**

This study found evidence that educated women lived closer to health centres than uneducated women (adjusted mean difference (adj MD) travel time −41 min (95 % CI: −50,–31)) in this community. Woman’s age was also associated with distance to the health centre. Women aged 15–20 years were more likely to live in a poor access area compared with women aged 21–30 years (adj MD travel time −11 min (95 % CI: −23, 0)), and with women aged 31–49 years (adj MD travel time −32 min (95 % CI: −47,-17)). There was no evidence to suggest that travel time to the health centre was associated with household wealth.

**Conclusions:**

Our main aim was to address the almost total lack of research evidence on what socio-demographic characteristics of women of reproductive age influence access to health facilities (in terms of physical distance). We have done so by reporting that our study found an association that women with no education and women who are younger live, on average, further away from a health facility in this rural Ethiopian community. While we have generated this valuable information to those who are responsible for providing maternal and child health services locally, to fully understand access in health care and to promote equitable access to health care, our study could thus be extended to other components of access and explore how our findings fit into the wider context of other factors influencing maternal health outcomes and utilisation of maternal health services such as antenatal care or delivery at health facility.

## Background

**Fig. 1:**
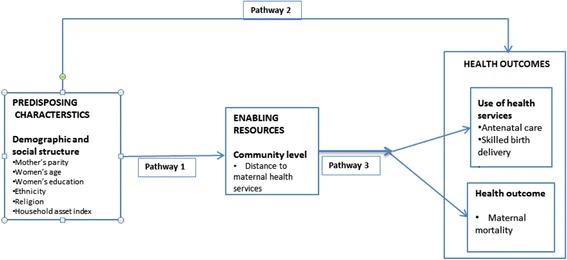
Conceptual framework to explore factors influencing access to maternal health services

‘Access to health care’ is one of the key concepts in health service research. Yet it has not been possible to formulate a definition to fit all contexts. Access has been defined as the “degree of fit” between health care clients and the health care system [1–3]. Whether individuals and groups actually gain access to health services depends on issues such as the affordability, acceptability, and physical accessibility of services [2]. Others propose an alternative approach to define access; for example, Donabedian (1972) proposes ‘proof of access’ to refer to ‘use of services’ not whether the facility exists [4]. Andersen (1995) proposed defining access as the “ability to use health services when and where they are needed” [5]. The Andersen model has three components: ‘enabling factors’ (resources that must be present so use of health services can occur [eg geographical proximity]); ‘need factors’ (level of disease or illness that triggers use); and ‘predisposing factors’ (socio demographic variables present before the onset of illness that describe individual tendency to use services [eg age, education, ethnicity]); [5] (see Fig. 1).

Socio-demographic ‘predisposing factors’ are often described as having an important effect on where people live [6, 7]. Yet very little work has been done to evaluate the association between socio-demographic characteristics and distance to health facilities, in remote African settings. The few studies that examined this association reported that factors such as age [8], ethnicity [9], and religion [10] are likely to influence poor access to health services. Other authors examined the importance of economic poverty in influencing access to health services and indicated that those who live furthest away from a health facility are most likely to be poor [6, 7]. Others have reported that distance to health facilities influences whether care is sought by those who need it, with utilisation of available maternal and child health services decreasing as distance from health facilities offering these services increases [11–15]. However, the risk and association may be substantially different in other settings such as remote mountainous areas in Africa.

Local evidence about the associations between socio-demographic characteristics and physical distance to health facilities is needed to understand the most disadvantaged groups in remote and rural populations. In this study we seek to examine the factors influencing physical access to health facilities in a rural area of Ethiopia, in women of reproductive age (15–49 years). The information will help to identify high risk women with poor access to health care. It will assist health service staff to understand constraints and design policies aimed at increasing uptake of reproductive and maternal health services.

## Methods

### Study site

The study was implemented from January–July 2010 in the Dabat HDSS in Dabat district, north-western Ethiopia. The Dabat Health and Demographic Surveillance Site (HDSS) consists of three urban and seven rural kebeles -the smallest administrative unit in Ethiopia. The population in the Dabat HDSS is currently 46,165 out of a total 182,000 population in the district. Farming, petty trade and animal husbandry are the main sources of income in the study area. During this study there was a single functioning health centre, located in Dabat town, serving the whole of Dabat district, with very few other options for health care within the district. The health centre provides both preventive and curative care including outpatient and inpatient care and serves as the first referral point for community based health care services. There are also eight satellite health posts in the district attached to the health centre. The health posts are the lowest-level in Ethiopian healthcare system and are staffed by two Health Extension workers providing the community with primarily preventive care including immunisation, family planning, maternal health services such as antenatal care and skilled birth attendant services. However, as in other parts of rural Ethiopia, only 26 % of pregnant women receive at least one antenatal consultation, and only 4 % deliver under the care of skilled providers [16]. Other care-seeking opportunities outside the district include Debark district hospital which is 40 km away from Dabat town and Gondar hospital – about 75 km away - which includes a medical school and functions as the referral hospital for the north Gondar zone. The study area is mountainous with poor road network. Off-road motorised transport is impossible because of terrain and the main form of transport is walking. A very small number of people use horses or donkeys for transportation; there are no motorised vehicles and bicycles cannot be used except in the semi-rural town of Dabat.

### Study design

In this study we adapted the Andersen’s behavioural model of health service use and Fig. 1 displays the conceptual framework used in this study. Pathway 1 in the figure illustrates this hupothesised relationship.

A household survey was conducted between January and July 2010. From a list of the rural households supplied by the Dabat Health and Demographic Surveillance Site, a computer generated random number sequence was used to select 208 households from each *kebele*. Women were eligible for inclusion in this study if they were resident in a selected household and aged between 15–49 years at the time of data collection.

### Data collection

Methods for data collection are detailed elsewhere [17]. In brief, during the household interview information on socio-demographic characteristics of the women including household assets, age, education level, occupation, religion, ethnicity, and parity was collected using personal digital assistants (PDA). Data collectors interviewed 2–4 households each day and ran a thorough check of each questionnaire for completeness and accuracy prior to leaving each household. If no woman was in the household to be interviewed, households were revisited one more time or as instructed by the study supervisor.

Each PDA was enabled with Global Positioning System (GPS) and at the end of the household interview; data collectors recorded the location (x-y coordinates) of the household. GIS was used to map locations of all households, the district health centre and the smaller health post facilities [17].

### Outcome and explanatory variables

The primary outcomes for this study were: travel time to the nearest health post and travel time to the district health centre from each household. Travel times were calculated using the “Cost analysis” module in the IDRISI Taiga GIS software package [18]. The methods are described in detail elsewhere [17]. In brief, the module requires two input layers of data. The first layer contains the target location (the health facilities), and the second layer contains the costs (in terms of the time spent walking) associated with moving through different geographical features in the study area to reach the target feature. Different features (e.g. walking up hills and mountains and traversing through water) are assigned different speeds. The output from the module is an image where each cell (pixel) in the image contains values of travel time required to traverse from that cell to the health post. A speed of 5 km/h was assigned for all walking routes, slopes greater than 30° were assigned a speed of 0.1 km/h. To validate the modelled travel time, actual travel time measurements from 40 village centres to the health centre were obtained as reference values and were compared to the estimated travel time. The comparison shows that mean actual and estimated travel time were very close (mean, 2hrs48mins vs. 2hrs42mins; standard deviation, 1hrs12mins vs. 1hrs6mins, respectively) and most of the differences between the reported travel time and estimated travel time had magnitude of less than 30mins. The result of validation has demonstrated good agreement and provided reassurance that that the estimated travel time is a valid measurement of geographical accessibility in rural Ethiopia. The findings of this validation are also consistent with previous studies which demonstrated this method of estimating geographical accessibility to be useful [19–22]. For example, Haynes et al. validated the use of estimated travel time based on distance to measure geographical accessibility by comparing a GIS-based travel-time model with actual driving time to service locations. Results were strongly associated for modelled and actual travel-time to health care service locations [23].”

The following variables were considered as exposures in the study: household asset index, woman’s education, woman’s age, parity. Construction of household wealth variables were based on a methodology proposed by Filmer & Pritchett [24] and extensive piloting in the local area. We included the possession of household items such as radio bicycle, size of their land, number of chicken, goats, cattle the household owned, and characteristics of the houses (roof type, toilet type, access to water, etc.). However, items that no households or all households own were not included in the analysis as they do not distinguish between poor and better-off. For instance, since no woman had a mobile phone, we excluded mobile phone from the items list. Thus only variables whose distribution varied across households were included. The asset variables were then either categorised into groups or dichotomised (i.e. new variables that take a value of 1 if the household owns the asset and 0 if the household does not own the asset were created). To test the correlation between variables, we used tetrachoric correlation as this was appropriate for our categorical variables. The asset index was then constructed using principal components analysis (PCA) to assign weights for asset variable. The first component, which accounted for 16 % variation captures the greatest variation in the original variables and was used to represent the assumed underlying construct of household wealth. Based on the first component a wealth score was generated, using a method described in Gwatkin et al. [25] and women in the sample were ranked in order of the asset index values for their households, and then they were divided into one of three equal sized terciles, ranking from the least poor to the poorest.

Woman’s education was defined by educational qualifications as reported by the women. To represent the fact that most women fell into the lowest end of the educational spectrum, the variable was dichotomised into ‘women with no education’ and ‘women with any education’. The age of a woman was defined to be her age at last birthday at the time of the interview. Women were grouped into three categories: ages 15–20 years, 21–30 years, and 31–49 years. Parity was defined as the total number of live births delivered by the woman and was based on data collected at household survey. The average parity was 4.2 while the maximum parity was 13. Based on this, parity was classified into four categories: a reference category (no live births), a second category that includes below average group (one to three live births), a third category where the average lie(four to five live births), and a fourth category that includes above average group(six or more live births).

### Ethical approval

This study was approved by the Ethical review committees of the University of Gondar and the London School of Hygiene and Tropical Medicine. Informed written consent was obtained from all participants in the study.

### Statistical analysis

In the univariable analysis, we first assessed the crude associations between each characteristic and travel time using simple linear regression, with results expressed as mean difference (MD) travel time between groups. To allow for potential confounding, multiple linear regression models were then fitted to examine whether associations for each socio-demographic variables were altered by adjusting for the other socio-demographic characteristics. The likelihood ratio test was used to obtain adjusted p-values. There was little evidence of within *kebele* or within household clustering of variables (most households contained only one woman in the 15–49 year age range), so multilevel models were not used. We did not adjust for parity when examining the associations between age and travel time to health facilities due to collinearity as the addition of parity to the model together with age lead to inflated standard errors. For this reason we decided against adjusting for parity when considering the impact of age on travel time. All analyses were performed in Stata SE 12.0 (Stata Corp LP, College Station, TX 77845, USA).

## Results

Between January and July 2010, 1,456 rural households have been selected and 1,451 were visited. Within those 1, 452, there were 1,422 women of reproductive age. Table 1 describes the characteristics of the 1,420 women who were included in the study. Of 1,420 women 530 (37 %) had received some formal education. The mean age of the women was 27 years with standard deviation 9 years; 495 (35 %) were categorised in the youngest age group (15–20 years) and 473 (33 %) in the oldest age group (31–49 years). The majority of women were parous with 964 (68 %) having had at least one child and 292 (21 %) having had six or more children. The population appeared almost completely homogenous in ethnicity and religion. The major ethnic group in the study population was Amhara (100) and 99 % of women were Orthodox Christians. The majority (68 %) were housewives and 63 % were illiterate.

**Table 1:** Characteristics of 1420 study participants in Dabat, rural Ethiopia

Variable	Categories	Number of women	Percentage of women (%)
Woman’s education (*N* = 1420)	No education	890	63
	Any education	530	37
Woman’s age (*N* = 1420)	15–20 years	495	35
	21–30 years	452	32
	31–49 years	473	33
* Parity (*N* = 1410)	0	446	32
	1–3	391	27
	4–5	281	20
	6–13	292	21
Occupation (*N* = 1420)	Housewife	964	68
	Student	298	21
	Farmer/other	158	11
* Household wealth (*N* = 1293)	Least poor	363	28
	Middle	459	36
	Poorest	471	36
Religion (*N* = 1420)	Orthodox Christian	1409	99
Ethnicity (*N* = 1420)	Amhara	1420	100

*There were 10 missing values for parity and 127 for household wealth

### Associations with travel time to Dabat health centre

On average, educated women lived 26 min closer to the health centre than uneducated women (MD travel time −26 min (95 % CI: −34, −20)) (Table 2). After adjustment for household wealth, woman’s age and parity, the association strengthened (adj MD travel time −41 min (95 % CI: −50, −31)). Woman’s age was also associated with travel time to the health centre. After adjusting for household wealth and women’s education in a multiple regression model, age was consistently associated with travel time to the health centre. Women aged 15–20 years had greater travel time than women aged 21–30 years adj MD travel time −11 min (95 % CI: −23, 0)), and women aged 31–49 years (adj MD travel time −32 min (95 % CI: −47,-17)).

**Table 2:** Associations between socio-demographic characteristics and travel time to Dabat Health Centre

Variable	Categories	N	Mean travel time (SD)	Unadjusted		Adjusted^a^	
				Mean difference (95 % CI), minutes	*P*-value	Mean difference (95 % CI), minutes	*P*-value
Household wealth	Least poor	363	2 h 43mins (1 h 5mins)	0	0.627	0	0.055
	Middle	459	2 h 38mins (1 h 3mins)	−4 (−13, 4)		−10 (−19,−1)	
	Poorest	471	2 h 41mins (1 h 8mins)	−2 (−11, 7)		−8 (−19, 1)	
Woman’s education	No education	890	2 h 50mins (1 h 7mins)	0	<0.0001	0	<0.0001
	Any education	530	2 h 23mins (56mins)	−26 (−34, −20)		−41 (−50, −31)	
Woman’s age	15–20 years	495	2 h 37mins (1 h 4mins)	0	<0.0001	0	<0.0001
	21–30 years	452	2 h 50mins (1 h 11mins)	13 (4, 21)		−11 (−23, 0)	
	31–49 years	473	2 h 32mins (58mins)	−5 (−13, 3)		−32 (−47, −17)	
Woman’s parity	0	446	2 h 30mins (1 h 1 min)	0	<0.0001	0	0.132
	1–3	391	2 h 53mins (1 h 10mins)	23 (14, 31)		10 (−2, 20)	
	4–5	281	2 h 40mins (1 h 7mins)	9 (0, 19)		−1 (−16, 14)	
	6–13	292	2 h 37mins (58mins)	7 (−2, 16)		2 (−14, 19)	

^a^Mean difference for household wealth and woman’s education and parity are adjusted for all variables in the model; mean difference for woman’ age is adjusted for household wealth and woman’s education only

On average, nulliparous women lived closer to the health centre than women with 1–3 children (MD travel time 23 min, (95 % CI: 14, 31)), with 4–5 children (MD travel time 9 min (95 % CI: 0, 19)) and with 6–13 children (MD travel time 7 min, (95 % CI: −2, 16)) respectively (Table 2). After having adjusted for woman’s age, woman’s education and household wealth in multiple regression models, there was no evidence to suggest that the mean travel times varied by parity (p-value 0.132).

There was little evidence to suggest that mean travel times differed by household wealth group based on results of both simple and multiple linear regression, with p-values 0.627 and 0.055 respectively (Table 2), although the association was found less likely to be due to chance in the adjusted model than in the simple model. However, the pattern was not consistent; the middle wealth group had lower mean travel time than both the poorest and least poor groups.

#### Associations with travel time to nearest health post

On average, educated women lived 9 min closer to their nearest health post than uneducated women (MD travel time −9 min (95 % CI: −12, −5)) (Table 3). After adjustment for household wealth, woman’s age and parity, the magnitude of the MD travel time increased, with an adjusted MD of −14 min (95 % CI: −19, −9) (Table 3). Women aged 15–20 years were less likely to live in a poor access area compared to women aged 21–30 years (MD travel time 5 min (95 % CI: 1, 9)) and more likely to live in a poor access area compared to women aged 31–49 years (MD travel time −4 min (95 % CI: −8, 0)) (Table 3). After having adjusted for household wealth and women’s education in the multiple regression model, it was apparent that older women were more likely to live closer to their nearest health post (adj MD travel time −8 min (95 % CI −13,-2), adj MD travel time −17 min (95 % CI −24,-10) for women aged 21–30 years and 31–49 years respectively; p-value <0.0001).

**Table 3:** Associations between socio-demographic characteristics and travel time to nearest health post

Variable	Categories	N	Mean travel time (SD)	Unadjusted		Adjusted^a^	
				Mean difference (95 % CI), minutes	*P*-value	Mean difference (95 % CI), minutes	*P*-value
Household wealth	Least poor	363	51mins (32mins)	0	0.882	0	0.335
	Middle	459	52mins (33mins)	0 (−4, 5)		−2 (−6, 2)	
	Poorest	471	51mins (30mins)	−1 (−5, 4)		−3 (−8, 1)	
Woman’s education	No education	890	54mins (34mins)	0	<0.0001	0	<0.0001
	Any education	530	47mins (26mins)	−9 (−12, −5)		−14 (−19, −9)	
Woman’s age	15–20 years	495	51mins (32mins)	0	0.0002	0	<0.0001
	21–30 years	452	56mins (34mins)	5 (1, 9)		−8 (−13, −2)	
	31–49 years	473	50mins (27mins)	−4 (−8, 0)		−17 (−24, −10)	
Woman’s Parity	0	446	47mins (29mins)	0	<0.0001	0	0.042
	1–3	391	56mins (34mins)	9 (5, 13)		8 (2, 14)	
	4–5	281	53mins (32mins)	6 (2, 11)		8 (1, 16)	
	6–13	292	47mins (28mins)	0 (−4, 5)		5 (−3, 13)	

^a^Mean difference for household wealth and woman’s education and parity are adjusted for all variables in the model; mean difference for woman’ age adjusted for household wealth and woman’s education only

On average, nulliparous women lived closer to their nearest health post than women with 1–3 or 4–5 children (MD travel time 9 min (95 % CI: 5, 13), MD travel time 6 min (95 % CI: 2, 11) for 1–3 and 4–5 parity groups respectively) but at a similar distance to women with 6–13 children (MD travel time 0.4 min (95 % CI: −4, 5)) (Table 3). After having adjusted for woman’s age in multiple regression models, the MD travel time reduced but remained statistically significant with adj MD travel time 8 min (95 % CI: 2, 14), adj MD travel time 8 min (95 % CI: 1, 16), and adj MD travel time 5 min (95 % CI: −3, 13) for 1–3, 4–5 and 6–13 parity groups respectively compared to nulliparous women; p-value 0.042). Similar to the results for travel time to Dabat health centre, there was little evidence to suggest that the mean travel times to nearest health posts were different in each household wealth group (p-value 0.882) (Table 3).

## Discussion

In rural Africa little is known about how travel time to health facilities is distributed among women of reproductive age and whether those who live far from a health facility have similar socio-demographic characteristics to those who live nearby. The paper fills this knowledge gap by reporting on the analysis of risk factors influencing poor access to health facilities in rural Ethiopia. Our findings demonstrated that both education and maternal age were significantly associated with travel time to both the main district health centre and the nearest health post. These associations persisted after adjustment for the other socio demographic characteristics considered in this study, namely, household wealth and parity. This finding is not consistent with finding of previous study in Ethiopia which indicate no association between age and use of maternal health services [26]. Reason for this inconsistent findings may be due to the fact that the previous study [26] measured the actual use of maternal services while we measured the physical accessibility of maternal health services. In the univariable model parity was a risk factor; however, the strength of evidence for this association reduced once the strong confounding effect of age was taken into account in the multivariable model.

Surprisingly, there was little evidence in our study to suggest that household wealth was associated with travel time to health facilities. Household wealth was not able to distinguish between women who were at high risk of living far from a health facility either before or after adjustment for other socio demographic characteristics. There are several potential reasons for our results. It may be that there truly is no variation in travel time to health facilities between the poor and less poor women. Or it could be that there is very little true heterogeneity in wealth in this setting. A limitation to this analysis is that although wealth variables were selected carefully, there might be misclassification of SES group leading to bias in the estimate of socio economic status or the wealth variable created may not have captured enough of the variability in asset ownership. Alternatively the use of the asset index approach as a measure of wealth may not have been a sensitive marker of inequality in geographical accessibility. In addition, there may be residual confounding, for example age due to misreporting or heaping may affect the result of the study.

It is interesting that similar results were observed for health centre and health posts, implying that it is the same women who have poor access to both, and these women are not receiving even basic primary health care provided by health posts. In theory health posts are supposed to reach more poor and isolated families in rural Ethiopia but this was not demonstrated in our study.

Our study found an association between living further away from a health facility and age and educational level. This was an observational study. Thus we cannot conclude with certainty that these determinants cause poor access to health facilities and the association of age and travel time to health facility can be partly due to unmeasured or residual confounding. However, it is plausible that women who are older and educated are more likely to live in areas that are more accessible to services. Older women may be more likely to be married and have the support of husbands to move closer to a service centre. Alternatively, younger women may feel healthy and have less desire to live close to a health centre or service centre. This is especially important because young women are known to have high risk of pregnancy related morbidity and mortality [27].

## Conclusion

Our main aim in this study was to address the almost total lack of research evidence on what socio-demographic characteristics of women of reproductive age influence access to health facilities (in terms of physical distance). We have done so by reporting that our study found an association that women with no education and women who are younger live, on average, further away from a health facility in this rural Ethiopian community. While we have generated this valuable information to those who are responsible for providing maternal and child Health services locally, given we have only focused on exploring the relationship between predisposing factors and enabling factors, very little can be said about health outcome and utilisation. Therefore, to fully understand access in health care and to promote equitable access to health care, our study could thus be extended to other components of Andersen model and explore how our findings fit into the wider context of other factors influencing maternal health outcomes and utilisation of maternal health services such as antenatal care or delivery at health facility. In addition, it is important to conduct in depth interviews to determine women’s perspectives on the barriers and facilitators including asking what sort of infrastructure they need, both individually and as a community to improving access to maternal health facilities in rural Ethiopia.
